# Targeting N-type calcium channels in young-onset of some neurological diseases

**DOI:** 10.3389/fcell.2022.1090765

**Published:** 2022-12-19

**Authors:** Flavia Tasmin Techera Antunes, Alessandra Hubner De Souza, Juliana Figueira, Nancy Scardua Binda, Vanice Paula Ricardo Carvalho, Luciene Bruno Vieira, Marcus Vinícius Gomez

**Affiliations:** ^1^ Department of Physiology and Pharmacology, University of Calgary, Calgary, AB, Canada; ^2^ Hotchkiss Brain Institute, University of Calgary, Calgary, AB, Canada; ^3^ Post-Graduate Program of Health Sciences, Faculdade de Ciências Médicas de, Belo Horizonte, Minas Gerais, Brazil; ^4^ Pharmacology Department, Universidade Federal de Ouro Preto, Ouro Preto, Minas Gerais, Brazil; ^5^ Pharmacology Departament, Universidade Federal de Minas Gerais, Belo Horizonte, Brazil; ^6^ Graduate Program in Health Sciences, Faculty Santa Casa BH, Belo Horizonte, Minas Gerais, Brazil

**Keywords:** Ca_v_2.2 channels, huntington disease (HD), multiple sclerosis (MS), migraine, N-type calcium channel, voltage-gated calcium channel (VGCC)

## Abstract

Calcium (Ca ^2+^) is an important second messenger in charge of many critical processes in the central nervous system (CNS), including membrane excitability, neurotransmission, learning, memory, cell proliferation, and apoptosis. In this way, the voltage-gated calcium channels (VGCCs) act as a key supply for Ca^2+^ entry into the cytoplasm and organelles. Importantly, the dysregulation of these channels has been reported in many neurological diseases of young-onset, with associated genetic factors, such as migraine, multiple sclerosis, and Huntington’s disease. Notably, the literature has pointed to the role of N-type Ca^2+^ channels (NTCCs) in controlling a variety of processes, including pain, inflammation, and excitotoxicity. Moreover, several Ca^2+^ channel blockers that are used for therapeutic purposes have been shown to act on the N-type channels. Therefore, this review provides an overview of the NTCCs in neurological disorders focusing mainly on Huntington’s disease, multiple sclerosis, and migraine. It will discuss possible strategies to generate novel therapeutic strategies.

## Graphical Abstract

**Figure:**
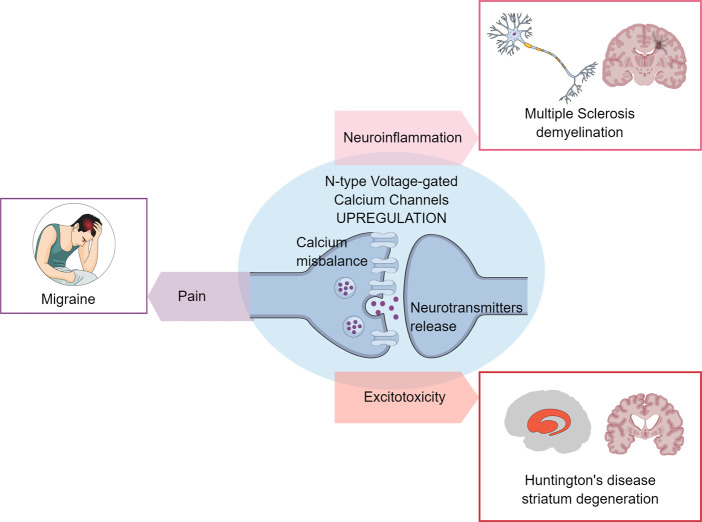
N-type calcium channels dysregulation and neurotransmission impairment Early-onset of neurological conditions, like Huntington’s disease, Multiple Sclerosis, and Migraine demonstrate harmful processes, such as neuroinflammation, neurodegeneration, and conditions like pain. N-type voltage-gated calcium channels are responsible for the exacerbated calcium influx that triggers neurotransmitter release.

## Introduction: Neuronal aspects of calcium signaling

The calcium ion (Ca^2+^) is a universal second messenger that regulates the activities of the eukaryotic cells (Brini et al., 2014). Ca^2+^ signaling mediates numerous physiological processes, including gene expression, excitation-contraction coupling, stimulus-secretion coupling, synaptic transmission, plasticity, and embryonic development (Mark et al., 2017; Islam, 2020). Likewise, Ca^2+^ is an important ion that is involved in several processes in the central nervous system (CNS), mainly neuronal excitability, and cell maintenance (Mark et al., 2017).

When it was discovered that Ca^2+^ was a carrier of cellular information, cellular control systems were developed, which maintain the concentration of Ca^2+^ within the cells (Brini et al., 2014). The Ca^2+^ concentration in the cytoplasm (100 nM free) is 20,000-fold lower than extracellular concentrations (2 mM). Thus, to maintain control of the Ca^2+^ gradient, the cells need to chelate, compartmentalize, or extrude it (Clapham, 2007). In the neurons, Ca^2+^ is tightly regulated by the protein systems that control cellular ion homeostasis and might be Ca2+ regulated themselves, both at the transcriptional and post-transcriptional level (Mellström et al., 2008). The Ca^2+^ influx occurs through the plasma membrane receptors and the voltage-dependent ion channels. The release of Ca^2+^ from the intracellular stores, such as the endoplasmic reticulum by the intracellular channels, also contributes to the elevation of cytosolic Ca^2+^ (Brini et al., 2014).

Different calcium channels within the plasma membrane are relevant for maintaining the Ca^2+^ gradient in the CNS cells. They include the voltage-gated calcium channels (VGCCs), the transient receptor potential (TRP) channels, and the calcium release-activated calcium (CRAC) channels (Gwack et al., 2007).

The VGCCs are key transducers of the membrane potential changes into the intracellular Ca^2+^ transients. The VGCCs are a family of membrane proteins that open in response to membrane depolarization, to permit the influx of Ca^2+^ along its electrochemical gradient (Zamponi, 2016). Early electrophysiological studies have detected distinct types of Ca^2+^ currents. The VGCCs comprise a range of different channels: L-type (Ca_v_1.1—Ca_v_1.4), P/Q-type (Ca_v_2.1), N-type (Ca_v_2.2), R-type (Ca_v_2.3), and T-type (Ca_v_3.1– Ca_v_3.3) channels (Nanou and Catterall, 2018).

Calcium channel dysregulation has been linked to several disorders, including Huntington’s disease (HD) (Raymond, 2017), Multiple Sclerosis (MS) (Enders et al., 2020), and Cephalic pain (Moutal et al., 2016a). Consequently, the calcium channels are considered important targets for therapeutic intervention.

### Voltage-gated calcium channels: Classification and structure

The VGCCs have been classified into two categories based on their activation threshold: high voltage-activated (HVA) channels that can open in response to higher membrane depolarization, and low voltage-activated (LVA) channels that can open in response to lower membrane depolarization (Catterall and Swanson, 2015). The HVA channels are subdivided, based on the pharmacologic and biophysical characteristics, into the L-, N-, P/Q-, and R types. The LVA channels are named T-type channels (Perez-Reyes, 2006). The HVA channels are heteromultimeric protein complexes, formed through the co-assembly of a pore-forming α1 subunit, and several auxiliary subunits (α2δ1-4, β1-4, and γ1-8), whereas the LVA channels appear to lack these ancillary subunits (Dolphin et al., 1999). The α1 subunit characterizes the channel subtype, whilst the ancillary subunits modulate the function and surface expression (Takahashi et al., 1987; Tanabe et al., 1987). Ten different α1 subunit subtypes fit into three families: the Ca_v_1 family encodes the L-type calcium channels, the dihydropyridine (DHP) sensitive L (long-lasting inward currents and they are composed of four different members (Ca_v_1.1–Ca_v_1.4). The Ca_v_2 family includes Ca_v_2.1 (P/Q-type inhibited by ϖ-agatoxin IVA, a toxin from the venom of the funnel web spider *Agelenopsis aperta*); Ca_v_2.2 (N-type inhibited by ϖ-conotoxin GVIA, a peptide toxin that is isolated from the fish-hunting cone snail *Conus geographus*); and Ca_v_2.3 (R-type inhibited by SNX-482, a toxin from the tarantula *Hysterocrates gigas*) (Mori et al., 1991; Dubel et al., 1992; Williams et al., 1992). The Ca_v_3 family covers up to three members (Ca_v_3.1–Ca_v_3.3 and all are blocked by Mibefradil and the piperidine-based molecules) (Mishra and Hermsmeyer, 1994; Uslaner et al., 2010; Choe et al., 2011).

### N-type calcium channel: General aspects

The N-type calcium channels (NTCCs) are members of the HVA Ca^2+^ channel family (Nowycky et al., 1985). They consist primarily of the α1B, α2δ, β1, β3, and β4 subunits (Catterall, 2000). The NTCCs have been identified in several types of cells and they are responsible for mediating the rapid Ca^2+^ influx into the synaptic terminal, triggering exocytosis, neurotransmitter release, synaptogenesis, and gene expression (Catterall, 1998). Pharmacologically, the NTCCs were first described and characterized in the presynaptic terminals of neurons, and are irreversibly blocked by ω-conotoxin GVIA (Olivera et al., 1987). To facilitate the effective excitation coupling within the II-III domain linker (synprint) of the NTCCs, the α1 subunit interacts with the proteins of the synaptic vesicle release complex, as syntaxin 1 and SNAP-25 (Sheng et al., 1994; Sheng et al., 1996).

The role of the NTCCs in nociception has been long established and implicated in the mediating neurotransmitter release in the primary neurons (Malmberg and Yaksh, 1994; Omote et al., 1996; Malmberg and Basbaum, 1998). The NTCCs are most abundant in the presynaptic terminals of laminae 1 and 2 in the dorsal horn of the spinal cord and are closely associated with synaptic vesicles that control the neurotransmitter release, such as glutamate, and substance P, from the primary afferent terminals (Snutch, 2005). The ω-Conotoxins are selective for the NTCCs and might block the substance P release in the spinal cord, showing antinociceptive in preclinical models of chronic pain (Schmidtko et al., 2010). Additionally, the Ca_v_2.2 channel blocker ω-conotoxin MVIIA is derived from Conus magus and was developed and licensed for chronic pain treatment as ziconotide (Prialt™) (Schmidtko et al., 2010). Concerning the inflammatory conditions, elegant work has shown that the NTCCs function changes in the rat dorsal horn during inflammation (Rycroft et al., 2007). Mice lacking the NTCCs have also shown decreased inflammatory pain and neuropathic pain signs (Saegusa et al., 2001).

It is well known that the excessive activation of the glutamate receptors, such as the N-methyl D-aspartate receptors (NMDARs, especially the NR1/NR2B-subtype), leads to many harmful outcomes, including the decrease of Ca^2+^ buffering, increased levels of the free radicals, mitochondrial membrane depolarization, and excitotoxicity (Lucas and Newhouse, 1957; Olverman et al., 1984). The literature has pointed out that NTCCs activity reduces survival, potentially by increasing the glutamate release, which might lead to NMDAR activation, increasing the Ca^2+^ levels, and leading to excitotoxic events (Sinor et al., 2000). Moreover, drug selection for the NTCCs has attenuated the neuronal injury in rodent cerebral ischemia studies (Valentino et al., 1993; Buchan et al., 1994).

### Huntington’s disease and the N-type calcium channel alterations

HD is an autosomal gene that is dominantly inherited. The neurodegenerative disorder is caused by an expansion of the CAG repeats in the first exon of the Huntingtin (*HTT*) gene (Saudou and Humbert, 2016). It results in the production of a long polyglutamine (PolyQ) fragment in the Huntingtin protein (HTT), inhibiting its activity, or merging into the mutant huntingtin (mHTT) aggregates (Scherzinger et al., 1997). These processes have led to the dysfunction and degeneration of the basal ganglia and the corticostriatal circuitry, reflecting the classical HD symptomatology in three main domains: motor, cognitive, and psychiatric (Beal, 1994; Vonsattel et al., 2011).

Particularly, the GABAergic medium spiny neurons (MSN) of the corpus striatum and the cortical pyramidal neurons (CPNs) are vulnerable to the mHTT expression (Pickrell et al., 2011; Vonsattel et al., 2011). Excitotoxicity contributes to the toxicity in HD, and it leads to neuronal dysfunction and death that is caused by the excessive activation of the glutamate-gated NMDARs, due to the enhanced glutamate release. This overloads the Ca^2+^ intracellular levels and the mitochondria energy failure (Coyle and Puttfarcken, 1993; Guo and Ma, 2021). In HD, this mechanism has primarily focused on the corticostriatal synapse, with the pre-and postsynaptic neuron receiving the most attention. The striatum receives the glutamatergic excitatory input from both the cortex and thalamus (Fonnum et al., 1981a; Fonnum et al., 1981b; Sepers and Raymond, 2014). The excitotoxicity hypothesis of the pathogenesis of HD is supported by the large entries and presence of high amounts of the glutamatergic receptors in the striatal neurons (Beal, 1994; Landwehrmeyer et al., 1995; Binvignat and Olloquequi, 2020). A variety of mouse models of HD support the excitotoxicity hypothesis. This fact is due to an overactivation of NMDAR that increases the response of the MSNs. When quinolinic acid was injected into YAC72 and YAC128 mice, there was a significant difference in the lesion size when compared to the wild-type mice, although as the disease progressed in these models, this phenotype did not persist in the older mice (Zeron et al., 2002; Graham et al., 2009). Furthermore, there is an increased glutamate level in the striatum of the YAC128 mice upon cortical stimulation (Dodart et al., 2000), although other studies suggest no such change in young YAC128 mice, while not yet displaying the behavioral or neuropathological features of HD (Götz et al., 2004).

Besides excitotoxicity, astrogliosis is one of the most prominent features found in the neuropathological examination of HD patients’ tissue (Vonsattel et al., 1985; Faideau et al., 2010). When it comes to neurodegenerative disorders, studies have shown that astrogliosis is a response to dysfunction or the death of neurons. There is a rise in the number of reactive astrocytes in the HD patient’s brains, as the disease grade increases (0–4). To properly maintain the synaptic function, there must be coordinated activity of the pre-and postsynaptic neurons but also of the astrocyte (Gray et al., 2019; Wilton and Stevens, 2020). The glutamate transporter is critical for regulating the glutamate levels at the synapse. This astrocyte transporter’s main role is removing glutamate from the synaptic cleft after it is released (Danbolt, 2001; Maragakis and Rothstein, 2001). The uptake of the main excitatory neurotransmitter of the CNS and its conversion into glutamine reduces its level in the synaptic space. *In situ* hybridization studies on brain tissue have shown that the excitatory amino acid transporter 2 (EAAT2) mRNA labeling decreases, and it correlates with HD severity and progression (Arzberger et al., 1997). Caudate and putamen show a decrease in the number of cells expressing EAAT2 mRNA. In addition, Grade 0 to Grade 4 tissues that are labeled with an EAAT2/GLT-1 antibody have revealed a grade-dependent decrease in protein levels by immunohistochemical analyses (Faideau et al., 2010). These results reveal that there is a loss of EAAT2 early in the disease process, which highlights the excitatory amino acid transporter 2 as an early component in the early development of HD (Gray, 2019). With excitotoxicity as one of the proposed toxic mechanisms in HD, due to the increased levels of glutamate in the striatal tissue that is thought to come from the corticostriatal inputs, the ability to efficiently capture glutamate from the synaptic space is of vital importance.

The development of the motor phenotype in R6/2 (express exon 1 of the human HD gene with ∼1,115–150 CAG repeats) and zQ175-KI (knock-in allele replaces the mouse HTT exon 1 with the human HTT exon 1 sequence, with ∼190 CAG repeats) models occurs early, for instance, through the increased excitability of the spiny projection neurons and the reduced number of primary dendrites, which seem to be partly non-progressive. However, other processes, like the increased Ca^2+^ release in the proximal dendrites, are marked by the enhanced involvement of the intracellular Ca^2+^ stores, potentially giving an apparent pathophysiological state (Sebastianutto et al., 2017).

As has been seen, disturbed Ca^2+^ signaling plays an important role in degeneration (Oikonomou et al., 2021). The intracellular Ca^2+^ level changes promote the altered neurotransmitter levels, mainly glutamate, which triggers excitotoxicity, although multiple pathogenic mechanisms like oxidative stress, neuroinflammation, and mitochondrial dysfunction, are involved in the pathogenesis of HD (Al-Nasser et al., 2022). When the Ca^2+^ demand increases, it also promotes the opening of the mitochondrial permeability transition pore (MPTP) and leads to apoptotic or necrotic cell death (Matuz-Mares et al., 2022).

In the CPNs from R6/2 mice, in the amplitude of action, the potentially induced Ca^2+^ transients were reduced in the presymptomatic and late symptomatic animals but were compensated by an increase in the decay times, which reflected an impairment in the Ca^2+^ efflux mechanisms. Nifedipine, a Ca_v_1.2 and Ca_v_1.3 VGCCs blocker, diminished the Ca^2+^ peak current, reducing the decay time, which reinforced it as a potential target for therapeutic intervention (Oikonomou et al., 2021). It is known that mHTT significantly decreased the Ca^2+^ threshold and was necessary to lead to the MPTP opening (Choo et al., 2004). Therefore, agents that reduce the Ca^2+^ influx and intracellular Ca^2+^ might modulate the progression of the disease (Oikonomou et al., 2021).

The VGCCs also regulate these processes. The zQ175-KI mice showed an increase in the synaptic vesicle release in the primary cortical neurons, with an increase in the Ca^2+^ influx at the presynaptic terminals, which was inhibited by blocking the NTCCs (Chen et al., 2018). The N-terminal fragments and the full-length huntingtin (HTT) also couple to the NTCCs (Swayne et al., 2005). In this line, the association between the mutant HTT affects the interaction of Ca_v_2.2 and the syntaxin 1A and Gβγ protein, which are their regulators (Silva et al., 2017). They also state that young (but no older) BACHD mice (bacterial artificial chromosome -BAC- expressing the human full-length mHTT, with 97 PolyQ repeats) exhibit an increased striatal glutamate release that is triggered by the increased Ca_v_2.2 Ca^2+^ current density and the plasma membrane expression but this finding was reversed by the NTCCs blockade. The same transgenic animal model, at the age of 3 and 12 months, showed an increase in the Ca_v_1.2 (L-type) protein levels in the cortex, along with an increase in the Ca_v_1.2 Ca^2+^ currents, and the new neuronal protection after the treatment with the Ca_v_1.2 blockers (Miranda et al., 2019). Another Ca_v_1. x blocker, Felodipine, has been shown *in vitro* to reduce the percentages of the cortical neurons with the mHTT exon 1 aggregates, and besides that, when injected intraperitoneally, the treated-B6HD mice (expressed the first 171 amino acids of mHTT, with 82 PolyQ repeats) displayed significant motor improvements, probably by inducing autophagy (Siddiqi et al., 2019). In the YAC128 mouse model (yeast artificial chromosome -YAC- expresses the full-length human HD gene, with 128 CAG repeats), the Ca_v_1.2 antagonist was mildly neuroprotective *in vivo* (Hong et al., 2015).

Long-term exposure to the protein aggregates (e.g., mHTT) can persistently activate microglia, and the degenerating neurons perpetuate a cycle of neurotoxicity and neurodegeneration (Hopp, 2021). Drugs that target Ca_v_1.2 or NTCC are neuroprotective and anti-neuroinflammatory, by decreasing the glutamate excitotoxicity and the activation of microglia, respectively. Probably, the subcellular location of each channel would contribute differently, once Cav1.2 is predominantly somatodendritic and a source of Ca^2+^ influx postsynaptically (Di Biase et al., 2011), while Cav2.2 is mainly presynaptic to participate in the vesicular release (Dolphin and Lee, 2020; Dolphin, 2021).

Under that scenario, current studies using animal venom toxins, particularly from the *Phoneutria nigriventer* spider, have shown higher interest in neurodegenerative disorders like HD (Antunes et al., 2022). CTK 01512-2, the recombinant peptide from the Tx3-6 native isoform, the NTCCs blocker, is displayed to improve the BACHD animal’s motor force, and the locomotor performance when delivered intrastriatal (Joviano-Santos et al., 2021), or intrathecal (Joviano-Santos et al., 2022) by inhibiting the neuronal apoptosis with a site-specific effect. In a chemical-induced HD model, CTK 01512-2 intrathecal was neuroprotective and improved the motor and cognitive behavior against the damage that was caused by an inhibitor of the mitochondrial enzyme succinate dehydrogenase (3-nitropropionic acid) (Antunes et al., 2021). These three studies have suggested that the protective effects of the peptide would be, at least in part, by decreasing the Ca^2+^ influx, the glutamate release, and consequently, the excitotoxicity. However, none of these studies has approached neuroinflammation.

### Multiple sclerosis and the N-type calcium channels alterations

MS is a neuroinflammatory condition that is marked by degenerative autoimmunity, which is directed against the nervous system (Lassmann et al., 2007). MS is chronic, where the inflammatory demyelination reflects the relapsing-remitting symptoms at the beginning, such as cognitive impairment, sensory disturbances, fatigue, motor weakness, walking impairment, problems with balance and coordination, bladder and sexual dysfunctions, and pain, although it progresses with an unpredictable course (Lassmann, 2018). It predominantly affects individuals in their early adult life, and has a huge impact functionally, financially, and on the quality of life (Thompson et al., 2018). Previous estimates of the occurrence of pain in MS patients have ranged from 50 to 300 per 100,000 people, and about 2.3 million people are estimated to live with MS globally (Thompson et al., 2018).

Pain is common in patients with MS, and it is estimated to occur in approximately 29%–86% of patients at the different stages of MS (Clifford and Trotter, 1984; Stenager et al., 1995). Pain is rarely observed in the early stages of MS. However, in advanced stages of MS, it is a symptom present in 50%–79% of patients (Brola et al., 2014). Several different types of pain have been described in patients with MS, while acute pain (usually paroxysmal) is present in 15% of patients but chronic pain is present in most cases (Warnell, 1991; Solaro and Uccelli, 2011). Because of this, the literature provides different classifications of pain types in MS. In 2008, O’Connor and colleagues proposed a classification of pain in MS into four categories: continuous central neuropathic pain, trigeminal neuralgia, musculoskeletal pain, and mixed neuropathic and non-neuropathic pain (i.e., headache) (O'Connor et al., 2008). The type of pain syndrome that occurs in MS is typically associated with the part of the nervous system that is involved (Racke et al., 2021). Central neuropathic pain has been reported as the commonest pain syndrome in MS and is associated with a primary CNS injury of the thalamus, or the parietal cortex, in which the projection areas for the sensory tract are located, and it is usually secondary to the lesions in the spino-thalamo-cortical pathways (Solaro and Uccelli, 2011). The estimated prevalence of central neuropathic pain is around 50% of MS patients (Solaro et al., 2013).

Neurodegenerative lesions commonly occur in the white matter of the brain and the spinal cord, and can also be observed in the brainstem and optic nerves (Frohman et al., 2006). There is various evidence that the neurodegenerative processes, like mitochondrial dysfunction, oxidative stress, and glutamate excitotoxicity, seem to play an important role in the pathogenesis of MS (Rajda et al., 2017). The initiation of the pathogenesis is characterized by the infiltration of the auto-reactive immune cells into the CNS, leading to inflammatory lesions, consequently inducing extensive demyelination and neurodegeneration (Noseworthy et al., 2000). The demyelination and axonal damage in the brain and the spinal cord lead to distinct mechanisms and central hyperexcitability. The lack of normal afferent impulses around the lesion causes alterations in the sodium and Ca^2+^ channels, which increase neuronal excitability (Brola et al., 2014). Ca^2+^ signaling has been implicated in many mechanisms of neurodegeneration under autoimmune inflammatory conditions (Fairless et al., 2014).

In MS, glutamate excitotoxicity has recently emerged as a potential mechanism that is involved in the pathogenesis of MS, and this might be a link of evidence between the inflammatory and neurodegenerative processes. Many studies have shown that glutamate levels are elevated in the cerebrospinal fluid of MS patients (Stover et al., 1997; Sarchielli et al., 2003), as well as in the acute MS lesions (Srinivasan et al., 2005). Several pathological mechanisms have been cited as a source and origin of glutamate excess, which is described in Table 1.

**TABLE 1:** The main causes of elevated extracellular glutamate in MS or EAE.

Phenome	Mechanism	Cells	References
Elevated extracellular glutamate	Increased Hemichannels of the Gap junctions	Microglia isolated from the primary mixed glial cell cultures from neonatal mice and peritoneal macrophages	Yawata et al. (2008)
	Increased system of the x_c_^−^ glutamate/cystine antiporter	Human monocytes and activated macrophages -microglia from rats with EAE and MS patients	Pampliega et al. (2011)
	Decreased expression of EAAT	Astrocytes/oligodendrocytes	Kostic et al. (2013)
	Increased ionotropic purinergic P2X receptor	Astrocytes from EAE rats	Vesce et al. (2007), Grygorowicz et al. (2016), Duan et al. (2003)
	Increased metabotropic Purinergic P2Y receptors	Astrocytes/Microglia from primary cultures (Cortices of newborn mice)	Vesce et al. (2007), Pascual et al. (2012)
	Increased TNFR1 receptor	Astrocytes from human and rat glial cell cultures	Bezzi et al. (2001)
	Increased mGlu receptors	Astrocytes cultured from the cerebral cortex of newborn rats	Bezzi et al. (1998)
	Ectopic VGCC distribution on the demyelinated axon	Rat neurons	Kukley et al. (2007)
Decreased Glutamate Reuptake	Decreased EAAT	Oligodendrocytes (from MS white matter)	Pitt et al. (2003), Werner et al. (2001)
	Loss of EAAT (reduced expression)	Activated microglia from the cortical lesions of MS patients	Vercellino et al. (2007)
	Increased mRNA but decreased EAAT protein levels	Forebrain and cerebellum of EAE rats	Mitosek-Szewczyk et al. (2008)
Glutamate receptor overexpression	Up-regulation of AMPA	Oligodendrocytes (from MS white matter)	Newcombe et al. (2008)
	Up-regulation of NMDA	Activated microglia and macrophages (from MS white matter)	Newcombe et al. (2008)
	Kainate agonism	Oligodendrocytes, Isolated from perinatal rat optic nerves	Matute, (1998)
	mGlu receptor overexpression	Activated microglia, astrocytes, and neurons (from MS patient sample)	Geurts et al. (2003)

EAAT: Excitatory Amino Acid Transporters; TNFR1: Tumor necrosis factor receptor 1; NTCC: N-type calcium channels; NMDA: N-methyl-D-aspartate; AMPA: α-amino-3-hydroxy-5-methyl-4-isoxazolepropionic acid; mGlu: metabotropic glutamate receptors.

Neurological disease, as well as its experimental model (experimental autoimmune encephalomyelitis—EAE), leads to motor, sensory, and cognitive deficits, where the pain is also a frequent symptom that is manifested as neuropathic pain (Constantinescu et al., 2011; Mirabelli and Elkabes, 2021). The NTCCs are the most studied in several pain types (Jurkovicova-Tarabova and Lacinova, 2019), and it is known that during MS and EAE, there is an overexpression of these channels in the active MS lesions and MS/EAE plaques (Kornek et al., 2000). The ectopic distribution of VGCCs in the axonal membrane might result in the Ca^2+^ influx, leading to axonal degeneration (Kornek et al., 2001; Enders et al., 2020). EAE is also aggravated by excessive glutamate transport and excitotoxicity (Pitt et al., 2000; Sheng et al., 2022). Therefore, P/Q and the NTCCs mainly regulate the glutamate release (Hess et al., 2010), and the blockade of these channels might contribute positively to ameliorating the MS/EAE symptoms and progression.

In a general way, the use of the NTCCs blocker CTK 01512-2 was able to prevent nociception, body weight loss, splenomegaly, MS-like neurological scores, impaired motor coordination, and the memory deficits that were induced by EAE (Silva et al., 2018). The NTCCs α(1B)-deficient mice were also less severely affected by the neurological symptoms of EAE (Tokuhara et al., 2010). However, further investigations are necessary about the link between the NTCCs and neuropathic pain, or the excitotoxicity and neuroinflammation in MS/EAE, as scarce studies are approaching this relation.

### Cephalic pain and the N-type calcium channels alterations

Migraine is a common disabling primary headache disorder (Bergman-Bock, 2018). Today, migraine is the third most prevalent disease in the world (Kowalska et al., 2016) and is also the third leading cause of disability in people under 50 years of age. The condition is three times more prevalent in women than in men (Bolay et al., 2015). The incidence is observed between the first years after two years of puberty, and the second in the years from 35 to 40 years old (Silberstein, 2004). This condition severely impacts patient functioning and the quality of life. It is usually underdiagnosed, and the treatment responses often remain poor, even after diagnosis (Cho et al., 2017). Migraine is divided into two main clinical subtypes: migraine with aura, and migraine without aura.

Before and during migraine attacks, the headache is accompanied by an increased sensitivity of the skin to touch (allodynia), as well as heat and cold (cutaneous allodynia), which indicates central sensitization (Bigal et al., 2008). According to the neurovascular theory of migraine, the activation of the trigeminovascular system (TGVS) and the release of numerous neuropeptides, mainly the calcitonin gene-related peptide (CGRP), are involved in headache pathogenesis. The TGVS can be activated by cortical spreading depression (CSD), a phenomenon responsible for the aura. The mechanism of CSD, stemming, in part, from the aberrant interactions between the neurons and glia, has been studied in models of a rare monogenic form of migraine with aura, the familial hemiplegic migraine (Kowalska et al., 2021). The pharmacological treatment of migraine has evolved over the years and nowadays, molecular therapies have assumed an important role in this disease (Demaagd, 2008).

Interestingly, while the presynaptic Ca_v_2 channels might be expected to drive the release of CGRP that is associated with migraine, the high-voltage activated and canonically postsynaptic Ca_v_1 channels and the low-voltage activated Ca_v_3 channels have both been found to regulate the CGRP release in the trigeminal ganglion (Amrutkar et al., 2011; Kowalska et al., 2021). Further, by correlating the genetic codependency of the Ca^2+^ levels, with the risk of migraine headaches, migraine can be linked to inherited hypercalcemia (Yin et al., 2017). Ca^2+^, as well as the potassium and sodium levels, are altered in the course of a migraine (Gargus, 2009), which is why scientists have been led to argue that migraine is channelopathy.

Calcium channel blockers exert their effect by blocking the Ca^2+^ influx into the vascular smooth muscle and cardiac muscle cells during membrane depolarization, leading to a reduction in blood pressure. Their main application is in the treatment of arterial hypertension and angina, and while used also for migraine pain relief therapy, they may themselves result in side effects, such as headaches and dizziness (Kowalska et al., 2021).

As calcium channel blockers are specific for different VGCCs and vary broadly in their pharmacologic effects, the development of therapeutics that selectively target these channels, which are centrally expressed and implicated in migraine pathogenesis, is of high research value. More studies are needed to understand the involvement of the VGCCs in the pathogenesis of migraine, and the search for headache-specific Ca^2+^ channel blockers remains.

Ca_v_2.2 has been extensively related as a mechanism for the treatment of pain, by controlling the neuropeptide release (Maggi et al., 1990), and more specifically CGRP, in the peripheral nervous system (Kress et al., 2001, like the TGs (Amrutkar et al., 2011). These channels are in a critical position to contribute to the headache-pain transmission, once they determine the nociceptive signaling from the dura to the trigeminal nucleus caudalis (Ebersberger et al., 2004). Importantly, the pretreatment of wild-type mice with the T- or NTCCs blockers has significantly reduced the duration of head-directed nocifensive behavior in a mouse model of headache, indicating that these channels are involved in the activation of the neuronal circuit underlying the migraine headache (Moutal et al., 2016a).

On another hand, the NTCCs activity is dependent on the axonal collapsin response mediator protein 2 (CRMP2) (Brittain et al., 2009; François-Moutal et al., 2015; Moutal et al., 2016a), and its cyclin-dependent kinase 5 (Cdk5)-phosphorylated form (François-Moutal et al., 2015). Thus, when inhibiting the CRMP2/NTCC interaction, it is possible to reduce the CGRP release (capsaicin-evoked) from the DRGs (Brittain et al., 2011; Piekarz et al., 2012; Ripsch et al., 2012). It is known that the CRMP2 expression is increased in the trigeminal branches in patients with chronic migraine (Guyuron et al., 2014). Therefore, targeting the NTCCs activity and inhibiting the CGRP release might contribute to the prevention of cephalic pain. In this sense, Moutal et al. (2016b) validated that (S)-lacosamide (an inhibitor of the CRMP2 interactions with the NTCCs) after an oral administration, reduced the Ca^2+^ influx in the TG neurons, diminished the evoked CGRP release in the dural afferents, and reversed cephalic and extra-cephalic cutaneous allodynia.

Narain et al. (2015) followed up on the case report of a 59-year-old woman, who had neuropathic lower extremity pain and severe migraine headaches, with 22 migraine days per month secondary to MS. Although her spasticity improved significantly with intrathecal baclofen, ziconotide was added to help her neuropathic pain complaints, and the treatment resulted in an improvement in both the lower extremity pain and a complete resolution of migraines, ensuing in zero migraine days per month (Narain et al., 2015).

Studies in mouse models of headache are needed to test the hypothesis that the N-type VGCCs in the dural afferent neurons are potential targets for anti-migraine therapeutics, in both abortive and preventive therapies. This will raise new insights into the contribution of Ca_v_2.2 to migraine pathophysiology, as well as the therapeutic potential.

## Summary and conclusion

**FIGURE 1:**
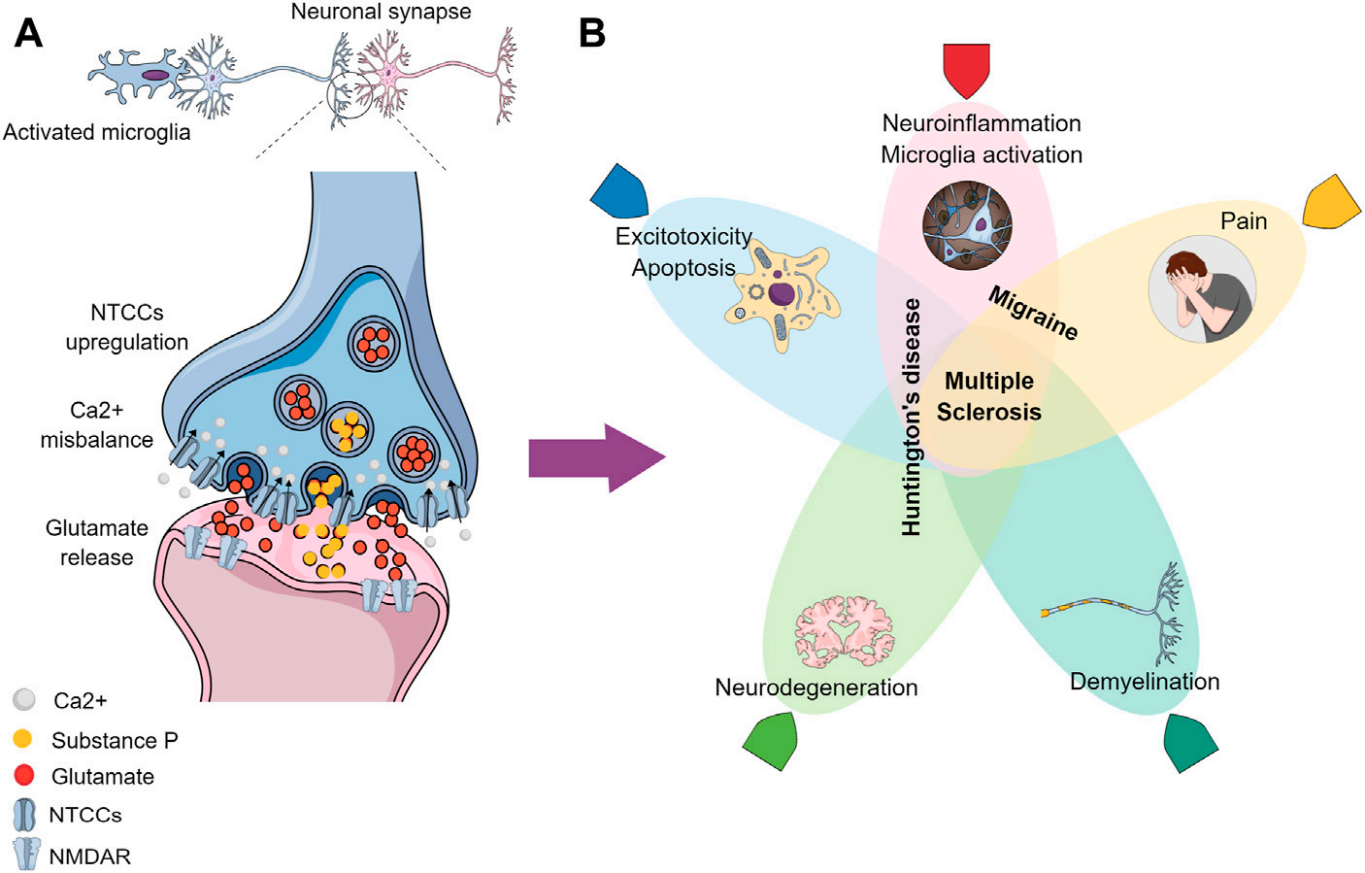
Graphical conclusion. **(A)** Activated microglia contribute to the increased neuronal excitability, where the NTCCs are presynaptically upregulated, causing a Ca^2+^ misbalance influx and an excessive neurotransmitters release (like glutamate and Substance P) in the synaptic cleft, which is propagated postsynaptically, due to the NMDAR excessive activation. **(B)** Processes/conditions resulting from the altered signaling have been demonstrated in **(A)**, where the overlapped zones represent similarities between the different neurological diseases: Migraine, Huntington’s disease, and Multiple Sclerosis.

Earlier-onset conditions like HD, MS, and migraine have been shown preclinically to share pathways where the NTCCs play an important role (Figure 1). The main studied biological NTCC blocker peptides are derived from animal venoms, like the *Phoneutria nigriventer* spider and the *Conus Magus* snail (Table 2). The topics that have been described previously highlight the gaps in the literature, where new approaches might be exploited toward the understanding of these neurological conditions. Posteriorly molecules that target these channels would need to clarify the effect in the pathophysiology before considering their use in clinical practice.

**TABLE 2:** Resume of studies about the NTCC blockers in Huntington’s disease (HD), Multiple Sclerosis (MS), and Migraine.

Author (year)	Compound tested - route	Study type - animal model	Disease model	Results
Joviano-Santos et al. (2021)	CTK 01512-2—Intrathecal	Preclinical—BACHD	HD	Improved the animal’s motor force and locomotor performance, reverted the muscle atrophy, protected the neurons in the spinal cord (but not in the brain) from death
Antunes et al. (2021)	CTK 01512-2—Intrathecal	Preclinical—3-nitropropionic acid (i.p.)	HD	Improved the motor and cognitive behavior, decreased glutamate in the CSF, and maintained the brain glucose uptakes
Joviano-Santos et al. (2022)	CTK 01512-2—Intrastriatal	Preclinical—BACHD	HD	Improved the animal’s motor force and locomotor performance, promoted neuronal preservation in the striatum and cortex
Silva et al. (2018)	CTK 01512-2—Intrathecal or intravenous	Preclinical—myelin oligodendrocyte glycoprotein (MOG, s.c.)	MS	Recovered spatial memory, improved motor coordination, and cognition reduced the mechanical and thermal allodynia, reduced inflammatory infiltrate, demyelination, leptin levels, the production of the pro-inflammatory cytokines, the astrocytic and microglial activation, and the brain glucose hypermetabolism, associated with an increase of the anti-inflammatory cytokine
Narain et al. (2015)	Ziconotide—Intrathecal	Clinical—Case study	Migraine	Complete resolution of the migraine headaches resulting in zero migraine days per month
Holden et al. (2022)	Ziconotide—Intrathecal	Clinical—Case study	Migraine	Potential therapeutic option for managing severe refractory migraine facial and cranial pain

HD is a neurological disease under genetic etiology, and preclinical studies have demonstrated that the involvement of the NTCCs was related to the interaction with the signaling complexes and the transduction cascades, leading to the disease development. However, the cellular loss that is caused by apoptosis and glutamate excitotoxicity might be improved by the NTCC blockers. Animal models of HD have a translational potential, as each different rodent’s model possesses an individual group of molecular and phenotypic characteristics, which allows one to use these differences to exploit the questions of interest and relevance (Pouladi et al., 2013). The VGCCs field is a little bit undertaken in the context of treatment for HD. It indicates that further research might be conducted to understand the individual role of the NTCCs while aiming at future pharmacological targeting.

Regarding MS, the NTCC blockers were revealed to be an effective strategy against induced demyelination and neuroinflammation, in addition to pain. Nevertheless, the data is too simplistic to determine how and where the NTCCs would act to impair or improve the cellular and molecular pathways in MS symptoms and progression. The authors of his review are in an initial state of the art, where the link between these components is not concrete, and needs additional investigations.

The NTCCs are a major determinant of nociceptive signaling and their activity controls the neurotransmitter release, like CGRP (Kress et al., 2001; Brittain et al., 2011; Moutal et al., 2016b). This finding places the channel in a critical position to contribute to migraine pain transmission. The Ca_v_2.2 blocker Ziconotide was shown to improve and prevent clinical pain migraine headaches (Narain et al., 2015; Holden et al., 2022). The exact mechanism was not investigated to elucidate the understanding of the underlying NTCCs in migraine, the specific spinal cord, or the brain areas, as far as Ca_v_2.1 is related to the pathology.

Although the function-specific channel biophysical properties seem to be clear, the splice variant expression patterns in these neurological conditions have not been studied at the moment. The present data is still scarce, which should be more robust, to add a translational value of biological drugs targeting the NTCCs for clinical management. Ziconotide was a drug approved in 2004 by the Food Drug Administration for severe chronic pain management. Its use is restricted to intrathecal administration because it does not cross the blood-brain barrier (Wie and Derian, 2022). Despite that, the recombinant form of the native peptide that is derived from *P. nigriventer* venom, CTK 01512-2, has been showing promising results against pain and neurological disease models in other injection routes besides intrathecal. This fact raises the peptide as a potential drug in the field when discovering new applications besides pain, although extensive research must be done to elucidate the NTCCs involvement properly in conditions like MS.
